# Long-term renal and cardiovascular outcome of living kidney donors: A single-center retrospective observation study

**DOI:** 10.3389/fmed.2022.966038

**Published:** 2022-09-14

**Authors:** Vincenza Colucci, Pasquale Gallo, Simona Simone, Luigi Morrone, Carlo Maria Alfieri, Loreto Gesualdo, Giuseppe Castellano

**Affiliations:** ^1^Struttura Complessa di Nefrologia e Dialisi, P.O.C. “S.S. Annunziata”, A.S.L. Taranto, Taranto, Italy; ^2^Nephrology Dialysis and Transplantation Unit, Department of Emergency and Organ Transplantation (DETO), University of Bari “Aldo Moro,” Bari, Italy; ^3^Department of Nephrology, Dialysis and Renal Transplantation, Fondazione IRCCS Ca' Granda Ospedale Maggiore Policlinico, Milan, Italy; ^4^Department of Clinical Sciences and Community Health, University of Milan, Milan, Italy

**Keywords:** living kidney donation, cardiovascular diseases, renal function decline, kidney transplantation, donation

## Abstract

**Background:**

The nephrectomy for donation reduces the renal parenchyma and glomerular filtration rate (GFR). It is important to understand the clinical consequences of kidney donation by a living donor.

**Methods:**

In this single-center, observational, retrospective study, we defined the renal and cardiovascular outcomes of living kidney donors. We analyzed data of 124 donors who donated at the Kidney Transplant Center (TC) of Bari between February 2002 and December 2018. Biometric data collected at visit 0, that is, at the time of the study of the donor candidate, and at visit 1, or rather at the last nephrological checkup (October-2018/August-2019) were compared.

**Results:**

An overall drop in GFR of 29 mL/min was observed over the analyzed period of 81+/-59 months. At visit 1, two donors developed chronic renal failure, including one in ESKD who underwent a kidney transplant. No relationship between age at donation and GFR drop was found. A trend toward an increase in obese people was reported; 28% of patients had compensated dyslipidemia and 35% were treated for hypertension. During the follow-up time, 3% had major cardiovascular events and 24% were lost to follow-up. One patient died.

**Conclusion:**

The age of the donor does not represent a basic element for reducing GFR or for the occurrence of major cardiovascular events. Furthermore, older donor candidates, in optimal health, should not be excluded from the donation. It is important to promote careful and timely follow-up of the donor, preventing the most common clinical consequences of nephrectomy, in consideration of the poor compliance of a large part of donors over the long-term post-donation period.

## Introduction

Kidney transplantation, particularly from a living donor, is the treatment of choice for most patients with end-stage renal disease (ESRD) (1). The superiority of the results achieved with kidney transplantation from living donors, associated with improved graft and patient survival compared with transplantation from a deceased donor, resulted in a progressive increase in this type of transplantation (2, 3). Living kidney donation, however, requires that healthy individuals voluntarily undergo major surgery with no physical health benefit to themselves. Although rare, perioperative mortality might occur during organ retrieval from living donors and have been estimated to occur in 0.03% of kidney donors (4, 5). Less serious perioperative risks are accepted and well-documented (5, 6). The kidney donation inevitably reduces functional renal parenchyma, determining a reduction of renal function. Additionally, in some cases, it can be associated with an increase in proteinuria, as well as with a rise in blood pressure (BP) greater than the general population (7, 8). These factors are associated with an increased risk of cardiovascular and all-cause mortality in the general population (9, 10).

In this retrospective single-center observational study, we aimed at defining the renal and cardiovascular outcomes of kidney donors for living donor transplantation.

## Methods

### Study design

We analyzed clinical and laboratory data of 124 donors undergone to a kidney donation at the Kidney Transplant Center (TC) of Bari during the period from February 2002 to December 2018. Biometric data collected at visit 0, that is, at the time of the study of the donor candidate, and at visit 1, or rather at the last nephrological checkup (from October 2018 to August 2019) were compared.

All procedures performed in studies involving human participants were in accordance with the ethical standards of the institutional and/or national research committee and with the 1964 Helsinki declaration and of the Declaration of Istanbul and its later amendments or comparable ethical standards. Given the observational and retrospective nature of the study, it was not necessary to obtain informed consent from patients. In any case, all the data were collected digitally, analyzed, and reported in the results in a totally and anonymous manner.

### Study population

The sample subject of the study was represented by a population of 124 donors, of Caucasian race, 71.7% female (W = 89; M = 35), with an average age at the time of donation of 54 years (54.3 years for women and 53.9 years for men) and with a distribution of 45.9% (*n* = 57) between 51 and 60 years. Twenty-two percent of patients (*n* = 28) aged between 61 and 70 years, 20.9% (*n* = 26) between 41 and 50 years, 7.2% (*n* = 9) between 31 and 40 years, 2.4% (*n* = 3) between 71 and 80, and 0.8% (*n* = 1) between 20 and 30 years.

It is interesting to note the degree of relationship between the donor and the recipient. In fact, 44.3% of donations (*n* = 55) were between mother and son/daughter, 14.5% (*n* = 18) between wife and husband, and 11.3% (*n* = 14) between sister and brother/sister. This confirms the previously described predominance of female sex among donors and is of high importance, considering that consanguine donors could have an increased risk of developing chronic kidney diseases.

The remaining transplants studied were 13.7% (*n* = 17) between father and son/daughter, 8% (*n* = 10) between brother and sister/brother, and 5.6% (*n* = 7) between husband and wife. Additionally, during the period considered, there was only one case of kidney transplant from a living between an aunt to her niece and another single donation between two young friends. During the analyzed period, there was also the first kidney transplant from a living donor, in crossover modality, of the TC of Bari between a husband in favor of his wife.

Of the 124 living donor transplants analyzed, 22.6% (*n* = 28) were performed in the preemptive modality. Regarding the recipient's nephropathy, 44 patients (35.5%) were affected by glomerulonephritis of which, 27 from IgA nephropathy, seven from focal segmental glomerulosclerosis (histological diagnosis), four from glomerulonephritis with histologically unspecified lesions, three from lupus nephritis, one from membranous-proliferative glomerulonephritis, one from extra capillary glomerulonephritis, and a case of granulomatosis with polyangiitis. Of the remainder, 36 (29%) were late referrals; 17 subjects (13.7%) had a diagnosis of congenital anomalies of the kidney and urinary tract (CAKUT). Autosomal dominant polycystic kidney (ADPKD) was the common pathology in 11 recipients (8.8%) while the remaining nephropathy was secondary to tubulointerstitial nephritis (4, 3.2%), nephrolithiasis (2, 1.6%), pre-eclampsia (2, 1.6%), chronic pyelonephritis (2, 1.6%), hypertension (3, 2.4%), cystinuria (1, 0.8%), renal tuberculosis (1, 0.8%), and bilateral renal cortical necrosis (1, 0.8%).

### Data collected

Blood and urine samples were obtained after an overnight fasting. Creatinine, urea, glomerular filtration rate (GFR) calculated according to the CKD EPI formula, 24-h proteinuria, fasting blood sugar, total cholesterol and triglycerides, calcium, and phosphorus were, therefore, collected and analyzed according to the in-center laboratory procedures.

These parameters were collected at the time of the evaluation of the donor candidate, before nephrectomy (visit 0), and compared with the same ones measured during the last nephrological check (visit 1). During the last outpatient visit, vitamin D and parathyroid hormone (PTH) were also measured in half of the sample.

The echocardiograms (in detail the ejection fraction, EF) of visit 0 and visit 1 were also compared; the latter performed by most of the population.

The analysis also compared the body mass index (BMI) and the medical history acquired at visit 0 and visit 1 with particular attention to the onset of cardiovascular events such as the appearance of arterial hypertension or the worsening of this if already existing, the finding of diabetes mellitus, as well as major cardiovascular events such as cases of ischemic heart disease or cardiac arrhythmias reported during follow-up.

### Statistical analysis

The distribution of variables was studied with the Shapiro-Wilk test of normality. Continuous variables with a Gaussian distribution were expressed as mean and standard deviation, while those with non-Gaussian distribution were expressed as median and interquartile range. Statistical comparisons were made with parametric and non-parametric tests, as appropriate based on the distribution of the variables. Categorical variables were expressed as an absolute numerical value and as a percentage. The multiple linear regression method was used to evaluate the relationship between delta GFR (GFR at visit 1—GFR at visit 0), as dependent variable and numerous independent variables. The effect of age at the time of donation on this relationship was evaluated by comparing the previous model with a mixed model, which included, with the same fixed effects, a random effect represented by the quartiles of age at the time of donation. Statistical significance was set at *p* < 0.05 values. Statistical analysis of the data was conducted using the statistical software R version 3.6.0.

## Results

The mean follow-up time was 81.5 +/- 58.9 months. In the analyzed period, it is possible to describe many kidney transplants from living donors <10 per year, except 2007 and 2008 with 10 and 11 donations, respectively. Subsequently, a significant reduction was observed in these until 2016 with a subsequent progressive increase, which led to a peak in 2018. In fact, in 2015, an outpatient clinic dedicated to living donor transplantation was instituted in our unit. In the group, a psychologist, nutritionist and some dedicated nurses were present who were really important for the study and follow-up process of the couples. This increased the couples being studied. In 2018, thanks to the increasingly better organization of the structure and the greater awareness raising and education about the donation by the team, the number of study couples increased significantly (61 vs. 17 in the 2017). This led to 19 live donor transplants being performed in 2018, thus doubling the annual rate of transplants compared to previous years.

The age of the patients was assessed at visit 0 and visit 1, as an absolute value and as quartiles of age. The age quartiles at visit 0 was I quartile: 26–48 years, II quartile: 49–54 years, III quartile: 55–60 years, and IV quartile: 61–72 years. The age quartiles at visit 1 were: I quartile 27–54 years, II quartile: 55–62 years, III quartile: 63–69 years, and IV quartile: 70–80 years.

### Follow-up of donors

The work showed a percentage of donors lost to follow-up that tends to increase over the years since the donation. In particular, in the first 2 years analyzed (2002 and 2003), the percentage of donors who no longer conduct checks is, respectively, 80 and 75%. The next 9 years, from 2004 to 2012, show an average of 33.7% donors lost to specialist follow-up. Donors who have undergone nephrectomy in the last 6 years result from the analysis to proceed correctly with outpatient appointments; therefore, there are no lost to follow-up, except one case (year of donation 2017).

Thirty patients up to 124 included in the study were lost to follow-up, resulting in an overall donor population analyzed of 94 patients.

The comparison of the principal anthropometric and biochemical data between visit 0 and visit 1 is summarized in Table 1.

**Table 1:** Comparison of the principal anthropometric and biochemical data between visit 0 and visit 1.

**Variables**	**Visit 0**	**Visit 1**
	**(*n* = 124)**	**(*n* = 94)**
AGE [mean (SD)], years	54 (9)	61 (10)
BMI [mean (SD)] kg/m^2^	25.3 (3.6)	25.8 (3.8)
CREATININE [median (IQR)] mg/dL	0.70 [0.60, 0.80]	0.95 [0.80, 1.10]
UREA [median (IQR)] mg/dL	33 [28, 38]	42[34, 48]
PROTEINURIA 24 H [median (IQR)], mg/24 h	57 [0.0,100]	128[0.00, 183]
GFR [median (IQR)] mL/min	98 [92, 103]	68 [60, 78]
GLYCEMIA [median (IQR)] mg/dL	91 [86, 9]	86 [78, 94]
TRIGLYCERIDES [median (IQR)], mg/dL	84 [61, 12]	102 [75, 127]

SD, standard deviation; IQR, interquartile range; BMI, body mass index; GFR, glomerular filtration rate estimated with CKD-EPI formula.

### Body mass index (BMI)

The change in the BMI of the donors between visit 0 and visit 1 was analyzed. The mean value of BMI at visit 0 was 25.3 ± 3.6 kg/m^2^, while at visit 1 was 25.9 ± 3.8 kg/m^2^, *p* = 0.29. The variation in BMI between visit 0 and visit 1 was also examined in different age quartiles, with reference to the age present at visit 0 and visit 1. A statistical significance was found only in the third-quartile of age (*p* = 0.004) in which the BMI increased from 24.9 ± 3.2 to 26.9 ± 3.2 kg/m^2^. No significant differences were found in the remaining quartiles.

Analyzing the distribution of donors in different categories based on BMI, it was found that in the pre-donation period there were two underweight subjects, 37 normal weight, a lot of overweight, and eight with class I obesity. At the last follow-up visit, their breakdown changes as follows: two underweight, 32 normal weight, 32 overweight, 12 with class I obesity, and two with class II obesity. No tests aimed to define a pre-diabetic status have been performed neither before nor after donation.

### Kidney function

Donors at visit 0 had a GFR value of 97 ± 11 ml/min (se 1.0; ci 2.1) while at visit 1 of 63 ± 15 ml/min (se 1.5; ci 3.1), *p* = 0.002.

In the observation period, an overall decrease in GFR of 29 ml/min is described.

The variation of the GFR was also studied in the age quartiles. Statistical analyses showed a statistically significant variation in the second, third, and fourth quartiles (in all cases *p* < 0.001) with changes in the GFR, respectively, from 102.3 ± 10 ml/min (se 1.5; ci 3.1) to 77.3 ± 12.3 ml/min (se 2.6; ci 5.5) in the second quartile; from 92.5 ± 9.9 ml/min (se 1.3; ci 2.5) to 66.7 ± 15.2 ml/min (se 2.4; ci 4.8) in the third quartile; and from 91.2 ± 5.9 ml/min (se 2.1; ci 4.9) to 62.4 ± 14.5 ml/min (se 2.7; ci 5.5) in the fourth quartile.

A multiple linear regression model was also applied considering dependent variable as the delta of GFR between visit 0 and visit 1 and as independent variables the following parameters recorded at visit 0: GFR, BMI, 24 h proteinuria, phosphorus, calcium, triglyceridemia, cholesterolemia, glycemia, hypertension, and ejection fraction. It is possible to observe how only the GFR at visit 0 significantly correlates (*p* 0.007; *R*^2^ 0.16) with the dependent variable (Table 2).

**Table 2:** Linear regression (dependent variable: delta GFR).

**Index var**	**Estimate**	**Std. error**	***T***	***P***	**Conf. low**	**Conf. high**
GFR. visit 0	0.3705	0.1339	2.7670	0.0071	0.1037	0.6372
BMI visit 0	0.3926	0.4467	0.8790	0.3822	−0.4974	1.2827
Prot-U visit 0	0.0228	0.0269	0.8468	0.3998	−0.0308	0.0763
Ca visit 0	2.9323	3.3074	0.8866	0.3782	−3.6579	9.5225
P visit 0	−0.4314	3.0450	−0.1417	0.8877	−6.4987	5.6360
TG visit 0	0.0378	0.0368	1.0270	0.3078	−0.0356	0.1112
CHOL visit 0	−0.0304	0.0450	−0.6766	0.5008	−0.1200	0.0592
GLYC visit 0	−0.2266	0.1514	−1.4966	0.1388	−0.5282	0.0751
Hypertension visit 0	−0.0246	5.4220	−0.0045	0.9964	−10.8282	10.7789
EF visit 0	0.0103	0.1880	0.0549	0.9564	−0.3643	0.3849
Observations: 85						

Model Fit: F_(10.74)_ = 1.424; p = 0.187; R^2^ = 0.161.

GFR, glomerular filtration rate calculated with CKD-EPI formula; BMI, body mass index; Prot-U, daily urinary protein excretion; Ca, Calcium; P, Phosphate; TG, triglycerides; CHOL, Cholesterol; Glyc, glycemia; EF, Ejection fraction.

The multilevel model with the same fixed effects and with the age quartile at visit 0 as a random effect has substantially overlapping coefficients to the previous model to indicate that the age quartile level does not significantly affect the model.

### 24 h proteinuria

An increase in 24-h proteinuria in kidney donors between visits 0 and 1 was demonstrated: 59.64 ± 65.2 mg/24 h (se 5.9; ci 11.8) at visit 0 and of 132.91 ± 165.31 mg/24 h (se 17.4; ci 34.6) at visit 1. Although the increase in this parameter is statistically significant, the 24-h proteinuria value of the donors observed at follow-up is not clinically relevant.

### Glycemia and lipid profile

The fasting blood glucose value of the donors at visit 0 was of 92.7 ± 10 mg/dl, while the blood glucose at visit 1 was 88.2 ± 14 mg/dl (*p* = 0.008).

Total cholesterol at visit 0 was 199 ± 36 mg/dl, while at visit 1 it was 186 ± 28 mg/dl (*p* = 0.007).

The triglyceride values were at visit 0 was 98 ± 51 mg/dl, while at visit 1 it was 114 ± 61 mg/dl (*p* = 0.04).

These changes are of no clinical significance.

### Calcium-phosphorus metabolism-related indexes

Changes in blood calcium and phosphorus values between visit 0 and visit 1 in kidney donors are not clinically and statistically significant.

At visit 1, the native vitamin D levels were 25 ± 10 ng/ml (*n* = 62).

At visit 1, PTH was 35.5 ± 15.8 pg/ml (*n* = 60).

The analysis of these laboratory parameters showed a picture of good overall metabolism.

### Health status

The clinical evaluation of living kidney donors, accompanied by the collection and updating of anamnestic data, made it possible to describe in this work the appearance of significant changes in the overall health status of the subjects studied. During the follow-up, only a case of breast carcinoma was reported.

Additionally, the study revealed the finding at visit 1 of two novel diagnosis of type 2 diabetes mellitus: in both cases, the patients were male and had a mute medical history at visit 0. One patient was in dual-therapy anti-hypertensive treatment, whereas the other patient had a revascularized ischemic heart disease and was treated for arterial hypertension in quadruple therapy and was dyslipidemia. He developed stage IV of CKD.

Twenty-eight percent of the donors in the sample showed dyslipidemia under pharmacological treatment, in all cases, except one, of new onset at visit 1 compared to 0. Additionally, 35.1% (*n* = 33) of the subjects analyzed in the study presented a diagnosis of arterial hypertension at visit 1. Of them, 25 patients were not hypertensive at visit 0. Specifically, it is possible to describe at visit 1 two cases in which the worsening of hypertension required the enhancement of antihypertensive therapy, from mono to dual therapy in one situation and from dual to triple therapy in the other.

Regarding the cardiovascular perspective, in addition to the incidence or persistence of arterial hypertension, three episodes of revascularized ischemic heart disease emerged from the analysis, which appeared in as many donors after 8 years of donation in one case and after 6 years in the other two. All three of these subjects at visit 0 had a mute medical history. In four other donors, arrhythmic events emerged at visit 1, in particular chronic or paroxysmal atrial fibrillation, and in one of these subjects, it was necessary to implant a pacemaker.

In two cases, the development of renal disease following kidney donation was found.

A 63-year-old man at visit 1, donor 14 years earlier, in 2005, of a kidney to his son. In the pre-donation medical history, there was no known pathology, yet the patient developed arterial hypertension in multiple therapy, dyslipidemia, type 2 diabetes mellitus, and in 2013 revascularized ischemic heart disease. To this complex clinical picture is added as the impairment of renal function with creatinine, at the last check conducted, of 2.4 mg/dl, urea 70 mg/dl, 24 h proteinuria of 1.2 g/day, and a GFR of 28 ml/min.

The other borderline case, unique in this sample examined, is that of a donor who in 2004, at the age of 59, underwent a preemptive donation in favor of her daughter, suffering from IgA-deposited glomerulonephritis. Over time, however, she developed end-stage chronic renal failure for which in 2018 the patient is registered on the waiting list for preemptive kidney transplants at the CT of Bari as a regional emergency. In the same year, a few months after enrollment, the donor received the transplant from a deceased donor.

In the sample of 124 donors examined in this work, three in the pre-donation study phase underwent renal biopsy for persistent macrohematuria. In these cases, nephroangiosclerosis was a unique finding, such as not to contraindicate the donation. Of the three patients, one was lost to follow-up; another, a donor of his son at the age of 60, was hypertensive at visit 0 and is still hypertensive at visit 1, on dual drug therapy; she has normal kidney function and good hemodynamic compensation. Finally, the third subject who underwent kidney biopsy donated his kidney to his daughter at the age of 48. At visit 0, he showed a mute history except for macrohematuria with good hemodynamic compensation. In this case, a cystoscopy was performed resulting normal. After 5 years of nephrectomy, at visit 1, he presents only a picture of dyslipidemia. Both of these two donors also presented at the visit 1 proteinuria in 24 h of <300 mg/day.

Concerning the survival of the donor population analyzed for a period of 81.5 months, there was only one death of a kidney donor to her child from unknown causes, which occurred in 2016 at the age of 75.

## Discussion

The awareness of the association between reduced renal function and the increased risk of cardiovascular mortality and morbidity (9, 11) places greater attention in assessing the clinical outcome of kidney donors. It is the responsibility of the medical staff to recognize and possibly quantify possible future risks to those patients. For this reason, candidates to donation undergo careful and scrupulous screening in the pre-donation phases.

The reduction in GFR observed in this population following nephrectomy could be accompanied by an increase in proteinuria, an increase in BP, as well as the appearance of other clinical complications indirectly associated with renal impairment (7, 8, 12), such as for example, type 2 diabetes mellitus or dyslipidemia. These conditions can occur in different times and ways than the population, similar in age and comorbidities, not subjected to nephrectomy.

The objective of this observational study is to verify the prognosis of a sample of kidney donors followed at the TC of Bari. The major limitation of this analysis, however, is represented by the lack of a control group composed by patients in which the donation program failed not for causes related to the potential donor.

According to the scientific evidence, about 10% of donors show, in the decade after donation, an increase in proteinuria (>300 mg/24 h) while in 12% of cases, there is a decrease in GFR <60 ml/min. The increase in proteinuria could be secondary to the hyperfiltration mechanism that the nephrons of the residual kidney undergo. This compensatory mechanism tends to increase over the years after nephrectomy and could finally lead to impaired renal function (8).

It would be important to define the prognostic value of proteinuria and GFR reduction in the donor population. In particular, for example, the use of GFR calculated with other methods (eGFRcys or mGFR) could give a more precise value of effective GFR in patient with < 90 ml/min.

Considering also that the mortality of donors is lower than that of the general population (13), the finding of these pieces of evidence in a percentage of subjects should not be interpreted as a deterrent to living kidney donation.

It is also important to consider how some consanguineous donors may have an unknown genetic predisposition to the development of kidney disease that can lead them to a condition of terminal uremia. In fact, consanguineous donors could share with the recipients some genetic traits and environmental factors. This could be the case of the only patient in our sample, a donor to her daughter suffering from IgA-deposited glomerulonephritis, who subsequently developed end-stage chronic renal failure and therefore underwent a preemptive kidney transplant from a cadaveric donor. In this case, no genetic test was available, so an eventual genetic predisposition could not have been excluded.

The remainder of our sample showed an overall decrease in GFR of 29 ml/min in the mean observation period of 81.5 months. It was impossible to compare this decrease in the filtration rate with that expected in the general population, equal in age and comorbidity of a control group. It should be considered that this reduction in GFR is affected by various comorbidities that have occurred over time, the aging of the population, as well as a very variable post nephrectomy observation time, from months to about 17 years. Furthermore, it was impossible to describe the trend of reduction of GFR per year with respect to the expected slope for aging because many donors analyzed did not perform regular annual laboratory checks. The analysis of the variation of the GFR by age quartiles, however, allowed us to observe how the reduction of this is not significantly influenced by the age of the donor. This result could have an important clinical value, confirming a good outcome of older donors.

Of note, according to literature, age should not be a parameter to exclude donors in the process of living kidney transplantation, but an estimation of lifetime ESRD risks for young individuals and late-life onset of end-stage kidney diseases must be addressed (14).

Of the 94 subjects studied, only in one case the presence of a GFR <30 ml/min was found at visit 1. This is a 63-year-old male patient, in post-donation follow-up for about 14 years, who developed major cardiovascular complications such as myocardial infarction, hypertension, diabetes, and dyslipidemia, which could be the cause of the development of the insufficiency pattern. Renal.

Concerning the appearance of proteinuria, in our sample, the mean value in the urine of 24 h at visit 1 is clinically insignificant (132.91 ± 165.31 mg/24 h); it can be added, however, that six donors presented at visit 1 a determination of 24 h proteinuria> 300 mg/day; of these, half has been in post-donation follow-up for more than 14 years and only one of them has a GFR <30 ml/min.

The remaining laboratory parameters examined showed how donors, regardless of their age group, retain an excellent metabolic compensation. Although there is a reduction in GFR, this is not associated with significant changes in calcium, phosphorus or with an increase in parathyroid hormone or a decrease in vitamin D. This confirms that donors do not always develop signs of chronic kidney disease (15–18).

Concerning cardiovascular risk, the analysis of the variation in BMI highlighted a redistribution in the various categories, which saw a reduction in normal weight subjects in favor of an increase in obese. The increase in body mass incidence represents an important cardiovascular risk factor as well as worsening of renal function indices (19).

With respect to major cardiovascular events, three subjects with ischemic heart disease and four with arrhythmic episodes are described in the entire population. There were two cases of new-onset diabetes mellitus, while 35% of the sample was affected by arterial hypertension and 28.7% by dyslipidemia.

The lack of the control group does not allow to compare these results with those expected in a general population. The literature does not show an increase in cardiovascular risk in donors, particularly in the first decade after nephrectomy.

On the subsequent observation period, there are currently no univocal data in this regard. In fact, it is possible that a reduction in GFR in these subjects may still lead to cardiovascular manifestations, but probably in a longer time (20).

The main elements on which to intervene in cardiological prevention are arterial hypertension, dyslipidemia, and BMI. The results of the work show that our donors therefore have risk factors that should be monitored clinically.

It is essential to promote adaptation to a correct lifestyle, awareness of the possible consequences, as well as a precise clinical-laboratory follow-up. The information must start in the study phase, before the donation, to continue during the outpatient checks. In fact, the periodic execution of blood chemistry tests and outpatient nephrological visits, repetition of annual cardiologic checks, monitoring of BP, and body weight are recommended (21, 22).

The work showed that there is a percentage of donors lost to nephrological follow-up equal to 24%. This value has a certain distribution of study time and therefore tends to increase with the passage of time from the donation. In the last 6 years, the subjects have all been compliant with the scheduled checks. If we also refer to cardiological follow-up, the subjects who have performed a cardiological visit in the last year are just 25.5%. Difficulty in accessing care is often reported by kidney donors; in fact, not all centers create a care path dedicated to the follow-up of the donor; the long waiting times for executing controls, not specifically nephrological, often discourages the execution.

The birth recently of surgery dedicated to living transplantation at the Kidney TC of Bari could justify the increase in compliance with the follow-up recorded in the most recent period. The awareness of the subjects toward the gift has been increased, as well as information about any risks and possible consequences. Couples are welcomed when they express their will to undertake this path, then guided in the preparation phase and conducted until the time of surgery. Attention is paid, in particular, to the donor, the aims are to identify any causes of clinical contraindication to the donation and probe the real motivation to perform the gesture.

## Conclusion

Living donor kidney transplantation is the best therapeutic choice for patients suffering from end-stage chronic renal failure as it reduces waiting times in the transplant lists, thus minimizing the patient's exposure to uremic status, limiting complications related to replacement treatments and in particular delays the onset of cardiovascular risk. Ultimately, it improves the recipient's outcome thanks to greater survival, higher quality of life, and cost reduction. However, the donor increases his self-esteem, undergoes a complete health checkup with subsequent periodic checks and improves the quality of life of his family.

In our population, it particularly emerged that age does not represent a discriminating element for reducing GFR or for the appearance of major cardiovascular events. This prompts us to affirm that donor candidates of advanced age, in the presence of an optimal state of health, ascertained in the pre-donation study phase, should not be excluded.

Furthermore, the importance of promoting a careful and timely follow-up of the donor emerges aimed at preventing the appearance of the most common clinical consequences of nephrectomy and the main cardiovascular risk factors such as an increase in BMI, BP, and dyslipidemia. The existence of a clinic figure dedicated to living donor study for the donation process can significantly increase the number of couples being studied and finally of donations. It is also necessary to take charge of the donor by a dedicated health team.

## Data availability statement

The raw data supporting the conclusions of this article will be made available by the authors, without undue reservation.

## Ethics statement

The studies involving human participants were reviewed and approved by University of Bari. Written informed consent for participation was not required for this study in accordance with the national legislation and the institutional requirements.

## Author contributions

LG and GC participated in research design. VC, PG, SS, LM, and CA participated in the writing of the paper. VC and PG participated in the performance of the research and in data analysis. All authors contributed to the article and approved the submitted version.

## Funding

This study was (partially) funded by Italian Ministry of Health—Current Research IRCCS.

## Conflict of interest

The authors declare that the research was conducted in the absence of any commercial or financial relationships that could be construed as a potential conflict of interest.

## Publisher's note

All claims expressed in this article are solely those of the authors and do not necessarily represent those of their affiliated organizations, or those of the publisher, the editors and the reviewers. Any product that may be evaluated in this article, or claim that may be made by its manufacturer, is not guaranteed or endorsed by the publisher.

## Abbreviations

ADPKD: Autosomal dominant polycystic kidney disease
BMI: Body Mass Index
CAKUT: Congenital anomalies of the kidney and urinary tract
EF: Ejection Fraction
ESRD: End-stage renal disease
GFR: Glomerular filtration rate
PTH: Parathyroid Hormone
TC: Kidney transplant center.
